# Prevalence of Human Papillomavirus in Adolescent Girls Before Reported Sexual Debut

**DOI:** 10.1093/infdis/jiu202

**Published:** 2014-04-16

**Authors:** Catherine F. Houlihan, Silvia de Sanjosé, Kathy Baisley, John Changalucha, David A. Ross, Saidi Kapiga, Jose M. Godinez, Ivana Bozicevic, Richard J. Hayes, Deborah Watson-Jones

**Affiliations:** 1 Clinical Research Department, London School of Hygiene and Tropical Medicine, United Kingdom; 2 Mwanza Intervention Trials Unit, Tanzania; 3 Unit of Infections and Cancer, Institut Català d'Oncologica, IDIBELL; 4 CIBER, Barcelona, Spain; 5 MRC Tropical Epidemiology Group, London School of Hygiene and Tropical Medicine, United Kingdom; 6 National Institute for Medical Research, Mwanza, Tanzania; 7 Collaborating Centre for HIV Surveillance, School of Medicine, University of Zagreb, Croatia

**Keywords:** human papillomavirus, prevalence, sexual debut, sub–Saharan Africa

## Abstract

***Background.*** Human papillomavirus (HPV) vaccines are recommended for girls prior to sexual debut because they are most effective if administered before girls acquire HPV. Little research has been done on HPV prevalence in girls who report not having passed sexual debut in high HPV-prevalence countries.

***Methods.*** Using attendance registers of randomly selected primary schools in the Mwanza region of Tanzania, we enrolled girls aged 15–16 years who reported not having passed sexual debut. A face-to-face interview on sexual behavior and intravaginal practices, and a nurse-assisted self-administered vaginal swab were performed. Swabs were tested for 13 high-risk and 24 low-risk HPV genotypes.

***Results.*** HPV was detected in 40/474 (8.4%; 95% confidence interval [CI], 5.9–11.0) girls. Ten different high-risk and 21 different low-risk genotypes were detected. High-risk genotypes were detected in 5.3% (95% CI, 3.5–7.8). In multivariable analysis, only intravaginal cleansing (practiced by 20.9%) was associated with HPV detection (adjusted odds ratio = 2.19, 95% CI, 1.09–4.39).

***Conclusion.*** This cohort of adolescent Tanzanian girls had a high HPV prevalence prior to self-reported sexual debut, and this was associated with intravaginal cleansing. This most likely reflects underreporting of sexual activity, and it is possible that intravaginal cleansing is a marker for unreported sexual debut or nonpenetrative sexual behaviors.

**(See the editorial commentary by Smith on pages 835–6.)**

Cervical cancer is the most common form of cancer in women in sub–Saharan Africa and the highest age-standardized incidence of cervical cancer in the world is found in East Africa at over 30.0 per 100 000 person-years [1]. This is compared with approximately 15.0 per 100 000 women worldwide, and 6.0 per 100 000 in North America [1]. Almost all cases of cervical cancer can be attributed to infection with 1 of 13 high-risk oncogenic genotypes of the human papillomavirus (HPV) [2]. Although limited in number, studies in women indicate that East Africa also has one of the highest global prevalences of HPV infection [3, 4]. Worldwide data consistently show that the prevalence of HPV is highest in younger women, most of whom will clear the infection within 10 months [5]. The early peak of HPV prevalence by age is explained by a rapid acquisition of HPV around the time of first sex in a previously unexposed, or immune-naïve, individual [6, 7].

The 2 available HPV vaccines, Gardasil (MSD) and Cervarix (GlaxoSmithKline Biologicals), cover the 2 most common cancer-causing HPV genotypes HPV-16 and -18. Additionally, Gardasil covers HPV-6 and -11, which, though low-risk for cervical cancer, are common causes of genital warts. HPV vaccination is most effective if administered prior to acquiring infection with these vaccine-related genotypes [8]. Data from North America and Europe support the assumption that most girls and women are HPV naive prior to first sex and that HPV is acquired quickly after sexual debut and with changes of sexual partner [7, 9]. However, there are no data on HPV prevalence in young women prior to reported sexual debut from high HPV-prevalence countries in sub–Saharan Africa, where sexual behavior, vaginal hygiene practices, and rates of underreporting of sexual debut may differ. Such data are important because they may identify modifiable risk factors for HPV infection, and because any planned national vaccination campaign will target young girls who are assumed to be HPV naive. Further, the World Health Organization (WHO) has recommended vaccination catch-up campaigns in older girls if a significant proportion can be assumed to be naive to vaccine genotypes [10].

To address the gaps in knowledge, we present baseline cross-sectional results from a cohort study in Tanzania that enrolled adolescent girls who reported that they had not passed sexual debut, and who were tested for vaginal HPV DNA. This study is the first to describe HPV prevalence in girls in sub–Saharan Africa who report no previous sexual intercourse, and the associations between reported sexual behaviors, vaginal practices, and HPV infection.

## METHODS

### Cohort Enrolment

The cohort was enrolled between January and August 2012 from previously prepared attendance lists of randomly selected government primary schools in 3 districts of the Mwanza Region in Tanzania. These lists, covering all primary schools in the districts, had been drawn up in 2010 to prepare for a trial of delivery methods for HPV vaccine [11]. The lists contained the pupil's name, date of birth, and which class they were enrolled in. Tracing information had also been collected on all girls. We enrolled eligible girls who had attended 1 of the 82 schools not selected to receive the vaccine.

Eligibility criteria included being aged 15 or 16 years at enrolment, enrolled in class 6 in 2010 in 1 of the selected schools, not pregnant (self-reported), planning to stay in the study area or able to travel to appointments, self-reporting never having had vaginal sex, and being willing to self-administer vaginal swabs. A subsample of 26 of the 82 schools were randomly selected and, in order to prevent stigmatization of girls who reported sex, at each of these 26 schools the first girl who reported ever having had sex was enrolled. An additional eligibility requirement for these girls was having passed sexual debut within the past 12 months.

### Ethical Issues

The London School of Hygiene and Tropical Medicine Ethics Committee and the Medical Research Coordinating Committee, Tanzania, approved the study protocol. Because all potential participants were considered minors in Tanzania (under 18 years old), written informed consent was required from a parent/guardian with subsequent participant informed assent. Consent for enrolment was taken before any assessment of previous sexual debut. The consent procedure involved separate face-to-face explanations of the study to parents and daughters by a study nurse, provision of written information, and time to ask questions. Individuals unable to read were consented in the presence of an independent witness who provided an additional signature. Individuals unable to write provided a thumbprint. Parents/guardians were compensated for their time with Tsh5,000 (approximately $3), and participants were provided with a toothbrush and toothpaste.

### Study Procedures

After enrolment, girls had a face-to-face interview with a female study nurse. Interviews were carried out in private at the participant's home, school, or local health center, depending on participant preference. Interviews were carried out in Swahili using a structured paper questionnaire, which had been translated and back-translated from English. Questions covered demographic and socioeconomic details, menstrual and vaginal hygiene practices, nonpenetrative sexual behaviors, and details of previous penetrative-sex frequency and partners. Interviews included colloquial terms for sexual behaviors that had been collected during focus group discussions with similar-aged girls. One self-administered vaginal Dacron swab was obtained after instructions from a study nurse, who remained in the room and provided verbal and positional hand guidance if necessary. Girls are being followed every 3 months for 18 months. We report results from the enrolment visit.

### HPV Detection and Genotyping

Immediately after collection, swabs were stored dry in cryotubes and placed into cold boxes with ice packs. They were submitted daily to the reference laboratory in Mwanza and stored at −20°C until they were shipped to the Catalan Institute of Oncology, Barcelona, Spain. HPV detection and genotyping were performed using the Linear Array HPV genotyping assay (Roche, CA) which detects 37 HPV genotypes (HPV-6, -11, -16, -18, -26, -31, -33, -35, -39, -40, -42, -45, -51, -52, -53, -54, -55, -56, -58, -59, -61, -62, -64, -66, -67, -68, -69, -70, -71, -72, -73, -81, -82, -83, -84, IS39, and CP6108). For this study, 13 HPV genotypes, HPV-16, -18, -31, -33, -35, -39, -45, -51, -52, -56, -58, -59, and -68, were classified high-risk [2]. Other genotypes were considered low-risk.

Briefly, DNA was extracted from the specimens by silica-gel-based methods (AmpliLute Liquid Media Extraction kit, Roche). Extracted material was amplified with the PGMY PCR system, and the generated amplicons were detected and typed by reverse-line blot reaction (Linear Array HPV Detection Kit and Genotyping Test, Roche). Polymerase chain reaction in this assay is based on a multiplex system, including human β-globin amplification primers. This provides an internal quality control for sample material. Specimens consistently negative for β-globin amplification were excluded because it was assumed that vaginal sampling was unsuccessful. All protocols were performed according to manufacturer's instructions: each step was performed in separate rooms and negative controls were used.

### Data Management and Statistical Methods

Questionnaire data were double-entered into OpenClinica LLC (Akaza Research, Waltham, MA), and analyzed using STATA V12.0 (StataCorp LP, College Station, TX). Baseline cohort characteristics were examined, and the prevalence of any HPV infection and prevalence of individual HPV genotypes were calculated. Household wealth was estimated using a score based on the number of possessions owned by the head of the household in which the participant currently resided. Median age of menarche in the cohort was calculated using Kaplan–Meier survival methods to account for those who had not reached menarche.

Factors associated with prevalent HPV infection (treated as a binary outcome for any HPV genotype vs none) were identified using logistic regression models. Age was considered an a priori potential confounder, and factors reaching *P* < .1 after adjustment for age were included in a multivariable model. Vaginal cleansing was investigated as a binary term for ever having cleansed, and as frequency of cleansing categorized as never, ≤2, and ≥3 times per day. All *P* values are from likelihood ratio tests.

## RESULTS

**Figure 1.**
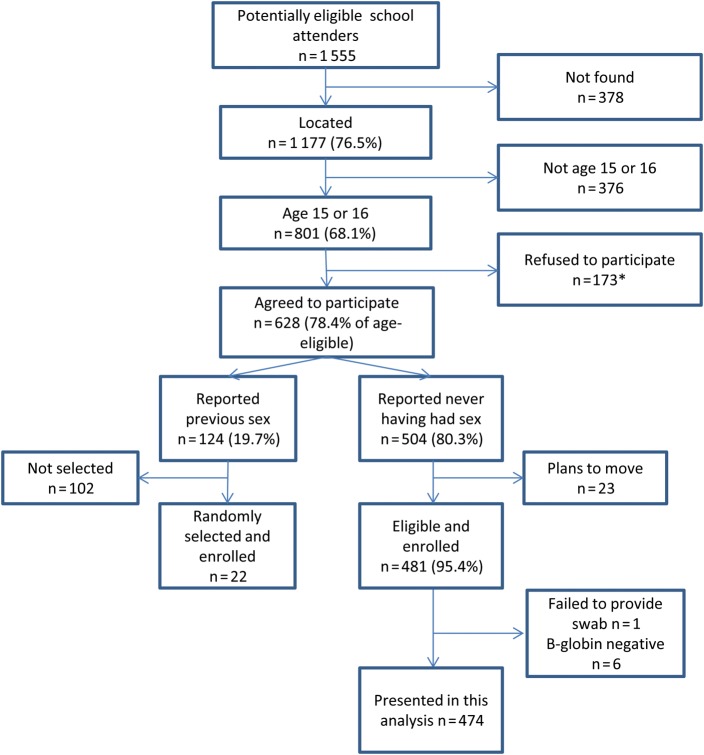
Flow diagram of cohort enrollment. *146 (84.4%) were parents and 27 (15.6%) were girls.

Of 1555 potentially eligible girls on the original school attendance lists, 1177 (76.5%) were located. From these, 801 (68.1%) were confirmed to be the appropriate age, and 628 (78.4%) consented to be screened for enrollment (Figure 1). Of those screened, 503 (80.1%) were eligible, selected, and enrolled, of whom 481 (95.6%) reported not having had sex. Those not enrolled either reported having had sex and were not randomly selected for inclusion (N = 102, 16.2%), or said they were planning to move out of the study area (N = 23, 3.7%). β-globin was detected in 495 (98.8%) of specimens. We excluded 6 participants from the analysis who provided β-globin–negative vaginal swabs, and 1 who did not provide a swab. We present HPV prevalence and associated factors from the remaining 474 girls who reported never having had sex, and the HPV prevalence in girls who reported having had sex within the past year.

### Cohort Description

Of the 474 participants who reported never having had sex, 225 were aged 15 (47.5%) and the remainder (52.5%) were 16 years old (Table 1). Only 11 (2.3%) reported ever having kissed, and 34 (7.2%) reported allowing a boy to touch their breasts. One girl reported touching a boy's penis, and another reported allowing a boy to touch her vagina. None reported oral or anal sex. The majority had passed menarche (N = 376, 79.3%). Median age of menarche in the cohort was 15 years (interquartile range, 14–15). One-fifth (N = 99, 20.9%) reported ever having cleansed inside their vagina; 78.8% (N = 78) using fingers and the remainder using a cloth (Table 2). Approximately half (53.5%) of those reporting cleansing used soap and water; the other half used water alone. One participant reported inserting a substance, tobacco powder, into her vagina.

**Table 1.** Analysis of Factors Associated With Detection of HPV in Adolescent Girls Who Reported Never Having Had Sex (n = 474)

Characteristic	No. (%)	HPV Positive No. (%)	Age-Adjusted Analysis		Ever Inserted	Ever Inserted
			OR (95% CI)^a^	*P* Value^b^	aOR (95% CI)^c^	*P* Value^b^
Overall	474	40 (8.4)				
Sociodemographic						
Age (years)						
15	225 (47.5)	14 (6.2)	1	.10	1	.10
16	249 (52.5)	26 (10.4)	1.76 (.89–3.47)		1.74 (.88–3.43)	
Current residence						
Urban	233 (49.2)	16 (6.9)	1	.13	1	.29
Rural	241 (50.8)	24 (10.0)	1.47 (.76–2.84)		1.43 (.74–2.79)	
Lives with						
1 or both parents	359 (75.7)	31 (8.6)	1	.23	1	.63
Other relatives or friends	115 (24.3)	9 (7.8)	0.84 (.39–1.84)		0.83 (.38–1.81)	
Husband	0		…		…	
Composite measure of household wealth						
High	113 (23.8)	11 (8.3)	1	.40	1	.91
Medium	228 (48.1)	18 (7.9)	0.84 (.38–1.85)		0.78 (.35–1.73)	
Low	133 (28.1)	11 (9.7)	0.91 (.37–2.19)		0.87 (.36–2.11)	
Current occupation						
Schooling or vocational training	329 (50.4)	25 (7.6)	1	.15	1	.33
Working	6 (0.8)	0	…		…	
Not working, not schooling	139 (29.3)	15 (10.8)	1.44 (.73–2.82)		1.40 (.71–2.77)	
Religion						
Christian	417 (88.0)	35 (8.4)	1	.21	1	.30
Muslim	43 (9.1)	3 (7.0)	0.85 (.25–2.88)		0.76 (.22–2.63)	
Other religion	8 (1.7)	2 (25.0)	3.28 (.63–17.06)		4.15 (.79–21.96)	
None	6 (1.3)	0	…		…	
Alcohol, drugs, or cigarettes (ever)						
No	473 (99.8)	40 (8.5)	…		…	…
Yes	1 (0.2)	0	…		…	
Sexual Behavior						
Kissed, ever						
No	463 (97.5)	40 (8.6)	…	…	…	…
Yes	11 (2.3)	0				
Breast touching, ever						
No	440 (92.8)	38 (8.6)	1	.53	1	.63
Yes	34 (7.2)	2 (5.9)	0.62 (.11–2.72)		0.71 (.16–3.10)	
Hand-genital contact with a boy, ever						
No	472 (99.6)	40 (8.5)	…	…	…	…
Yes	2 (0.4)	0				
Oral sex, ever						
No	474 (100)	40 (8.4)	…	…	…	…
Yes	0	0	…		…	
Menstruation						
Passed menarche						
No	98 (20.7)	7 (7.1)	1	.24	1	.84
Yes	376 (79.3)	33 (8.8)	1.73 (.88–3.43)		1.09 (.46–2.58)	
Sanitary item used during menstruation^d^						
Cloth or paper	171 (36.1)	11 (6.4)	1	.33	1	.34
Underwear	77 (16.2)	8 (10.4)	1.67 (.64–4.33)		1.69 (.65–4.41)	
Sanitary napkin	128 (27.0)	14 (11.0)	1.90 (.83–4.84)		1.78 (.77–4.13)	
Intravaginal practices						
Ever cleansed						
No	375 (79.1)	26 (6.9)	1	.03	1	.03
Yes	99 (20.9)	14 (14.1)	2.19 (1.09–4.39)		2.19 (1.09–4.39)	
Ever inserted						
No	473 (99.8)	40 (8.5)	…	…	…	…
Yes	1 (0.2)	0	…		…	

Abbreviations: aOR, adjusted odds ratio; CI, confidence interval; HPV, human papillomavirus.

^a^ ORs adjusted for age as a priori confounder.

^b^ *P* value from likelihood ratio test.

^c^ ORs adjusted for age and ever cleansed.

^d^ Of those passed menarche.

**Table 2.** Intravaginal Cleansing and HPV Prevalence

Characteristic	No. (%)	HPV Positive No. (%)	aOR (95% CI)^a^	*P* Value^b^
Frequency of cleansing				
Never	375 (79.1)	26 (6.9)	1	.01
≤2 per day	61 (12.9)	5 (8.3)	1.21 (.44–3.28)	*P trend* = *.002*
≥3 per day	38 (8.0)	9 (23.7)	4.03 (1.72–9.45)	
Cleansed with				
Water	46 (46.5)	5 (10.9)	1	.39
Soap + water	53 (53.5)	9 (17.0)	1.67 (.51–5.39)	
Cleansed using:				
Fingers	78 (78.8)	13 (16.7)	1	.13
Cloth	21 (21.2)	1 (4.7)	0.25 (.03–2.04)	

Abbreviations: aOR, adjusted odds ratio; CI, confidence interval; HPV, human papillomavirus.

^a^ Adjusted for age as a priori confounder.

^b^ *P* value from likelihood ratio test.

### HPV DNA Prevalence

**Figure 2.**
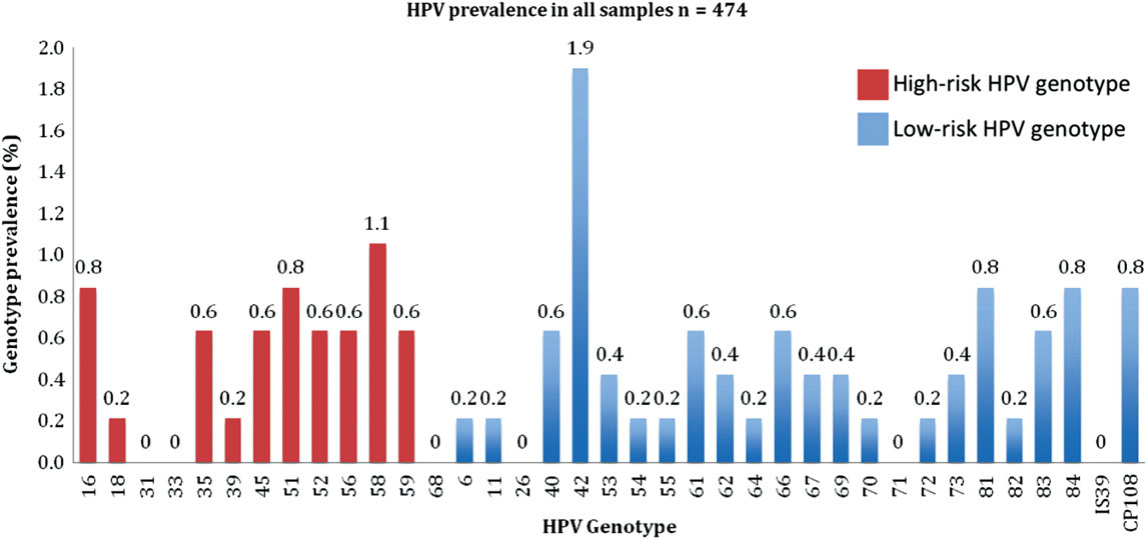
Human papillomavirus (HPV) genotypes detected in girls reporting never having had sex (n = 474).

HPV DNA was detected from vaginal swabs in 40 of 474 girls who reported not having passed sexual debut, giving a prevalence of 8.4% (95% confidence interval [CI], 5.9–11.0). In total, 24 (5.1%, 95% CI, 3.1–7.0) girls had a high-risk HPV genotype. Multiple genotype infections were detected in 20 (4.2%, 95% CI, 2.4–6.0) girls, comprising 50.0% of those with HPV DNA. The 2 most commonly detected genotypes were HPV-42 and HPV-58, with a prevalence of 1.9% (N = 9) and 1.1% (N = 5), respectively (Figure 2). The prevalence of infection with any of the vaccine genotypes HPV-6, -11, -16, or -18 was 1.6% (95% CI, .5–2.7).

A total of 22 eligible girls who reported having passed sexual debut within the last year were enrolled from 26 randomly selected schools. HPV DNA was detected in 7 (31.8%, 95% CI, 10.7–53.0) of these 22 girls, significantly higher than in those who reported never having had sex (*P* < .01), and high-risk HPV was detected in 22.7% (95% CI, 3.7–41.7, N = 7). Though the numbers involved were small, their demographic profile was similar to that of girls who reported no previous sex (data not shown).

### Associations With HPV

In univariable analyses of 474 girls who reported never having had sex, there was weak evidence that being age 16 years was associated with HPV detection (odds ratio, 1.76, 95% CI, .89–3.47; *P* = .10). Only the reporting of ever having practiced intravaginal cleansing was associated with HPV DNA detection in univariable analysis, and this persisted after adjustment for age (adjusted OR [aOR], 2.19, 95% CI, 1.09–4.39; *P* = .03) (Table 1). Furthermore, there was strong evidence of a dose-response relationship between cleansing frequency and HPV DNA detection (per unit increase in cleansing frequency category after adjustment for age, aOR, 1.54, 95% CI, 1.17–2.03; *P* = .002). However, among girls who reported cleansing, there was no evidence of an association of HPV detection with method (fingers vs cloth), or substance used (water alone vs soap) (Table 2).

## DISCUSSION

In the first study to examine the epidemiology of HPV in girls from sub–Saharan Africa who self-reported never having engaged in vaginal sex, the prevalence of HPV was surprisingly high (8.4%) based on the testing of self-administered vaginal swabs. This is higher than in 2 previous studies among women who reported no previous sex: 1 study of 130 Swedish women aged 10–25 years reported a prevalence of 1.5%, and 1 longitudinal study of American women aged 18–20 years reported that 1.7% of specimens were found to have HPV DNA [7, 9]. A further 3 small studies in Sweden, Denmark, and Australia found no HPV DNA in cervical or vaginal specimens from 15, 30, and 55 women, respectively, aged between 13 and 41 years, who reported no previous sex [12–14].

Previous studies have confirmed vaginal sex as the predominant method for acquisition of cervical HPV infection in women, as demonstrated by rapid HPV acquisition after reported sexual debut, and a significant increase in the risk of HPV infection with increased number of partners and with high-risk partners [13, 15]. Lack of disclosure of previous sex is, therefore, the most likely explanation for the relatively high HPV prevalence observed in our study. Previous research in adolescents in different world regions consistently illustrates underreporting of sexual behaviors [16, 17], and studies conducted in the same geographical area of Tanzania as this study have demonstrated underreporting of sex in girls of similar ages, and in older, sexually active women [16, 18]. In young females in this region of Tanzania in which the median age of reported sexual debut is 16 [19], potential consequences of disclosure of sex under the age of 16 include expulsion from school, physical punishment, and social exclusion [20]. However, irrespective of reporting errors, finding HPV in specimens collected from girls who report no previous vaginal sex remains important. Vaccine efficacy is highest in those who have not previously been exposed to the vaccine-related HPV genotypes, and age of vaccination is based on the assumption that girls are HPV negative prior to self-reported sexual debut [21]. Our data reinforce WHO recommendations that vaccination should be targeted at younger girls [10] and suggest that the vaccination target age should be several years before the median age of self-reported sexual debut in the population.

Alternative explanations for the presence of vaginal HPV DNA in girls and women who report no previous sex include mother-to-child transmission (MTCT) of HPV, nonpenetrative sex practices, or transmission via fomites [22]. MTCT is the primary mechanism for HPV acquisition in cases of respiratory papillomatosis in children. However, in well-conducted prospective studies, MTCT of genital HPV types was infrequent and most often transient [23, 24]. Transmission through nonpenetrative sex practices such as hand-genital or oro-genital contact has been described in exclusively homosexual women in the United States who report never having had sex with a man (4/21 women, 19%) [25]. However, only 2 participants in our sample reported these practices, and neither had detectable vaginal HPV DNA.

In the age-adjusted analysis in our study, disclosure of ever having practiced intravaginal cleansing was associated with more than doubled odds of prevalent HPV. Furthermore, a positive dose-response relationship was seen with increasing frequency of vaginal cleansing. Commonly, women use intravaginal practices (IVP) to manage menstruation, as part of their sexual practice (altering vaginal lubrication or tightness), and to improve genital hygiene. IVP includes both cleansing inside the vagina with water, soap, or other products, and the insertion of products into the vagina (eg, pulverized herbs) [26]. IVP is a common practice in many parts of sub–Saharan Africa [27], and has been reported in 96% of women working in bars and guesthouses in Tanzania [26]. In that population, many women reported initiating IVP at the time of menarche or when they were given instruction about sex or marriage [28], and in our study, 20.9% of girls reported intravaginal cleansing. IVP has been associated with increased risk of HIV acquisition in sex workers in Kenya, and with a doubled risk of acquisition of a new sexually transmitted infection (STI) in a prospective study of adolescent girls in the United States [29, 30]. With specific relevance to HPV, a cross-sectional study of 312 adolescent girls in the United States found that vaginal cleansing in the past 90 days was associated with a doubling of the odds of cervical HPV infection [31].

HPV has been detected in fingernail and fingertip specimens of young sexually active women in the United States, as well as on toilet seats in several European airports, and on surfaces in a sexual health clinic in the United Kingdom [32, 33]. HPV's viability to cause infection of animal cells after desiccation has been demonstrated in a United Kingdom laboratory setting [34]. In East Africa, HPV infection in women is highly prevalent [3, 35, 36] and therefore an adolescent girl who performs intravaginal cleansing may theoretically self-infect with HPV either from her own external or extragenital sites, or from objects such as cloth, taps, or water buckets contaminated from other household members. Acquisition of HPV from household members may be direct, via the insertion of shared cloths, or indirect via contaminated fingers. A similar explanation has been postulated for the presence of vaginal *Trichomonas vaginalis* in Zambian girls aged 13–16 who reported no previous sexual contact [37]. Intravaginal cleansing may additionally increase the risk of HPV infection through vaginal mucosal abrasions, allowing HPV to access target basal membrane cells, or through alteration of the vaginal microbiome, which has been associated with increased risk of other viral STIs, including HIV [38].

An important and likely explanation for the observed association between intravaginal cleansing and HPV is that intravaginal cleansing may be a marker of unreported sexual debut or nonpenetrative sex behaviors. Girls may be more likely to start vaginal cleansing after sexual debut because they believe that this may prevent pregnancy or STIs, relieve STI-related symptoms, or because they have been taught that this practice is appropriate for sexually active women [27]. Increased frequency of cleansing has been associated with higher number of sex acts and/or partners [26], as has HPV acquisition [7], which may explain the dose-response relationship between intravaginal cleansing and HPV infection seen in our study.

Multiple HPV-genotype infections were as common as single genotype infections in this study, consistent with observations in adolescent girls in previous studies in the United States [31, 39]. More than half of the girls with HPV were found to have a high-risk genotype, also consistent with the literature on sexually active adolescent girls or young women in Denmark, Canada, and Tanzania [13, 35, 40]. Of the 40 girls who had HPV, only 6 (15.0%) had 1 of the 4 HPV genotypes included the currently licensed vaccines, and only 4 (10.0%) had 1 or both of the 2 high-risk genotypes in the vaccine.

Major strengths of this study include the interview method, in which the questionnaire included local age-specific colloquial terms for sexual practices, and was administered by trained nurses experienced in adolescent sexual behavior research. In addition, vaginal swabs were self-administered but collection was directly observed by nurses. Self-administered vaginal swabs in a previous local study (median age 17) demonstrated collection of epithelial cells in 233/244 (95%) of swabs [41], and 99% of our specimens contained β-globin, indicating successful sampling. Studies have shown a strong correlation for HPV detection between clinician-collected and self-administered vaginal swabs [42, 43]. Finally, the method used for HPV detection and genotyping, Roche Linear Array, has been proven in large studies to be highly sensitive [44, 45].

One of the limitations of this study is that the enrollment list came from primary schools. Although primary school attendance is a legal requirement in Tanzania, and has been reported as 97% [46], it may be lower in rural populations and in upper-primary-school years (from which this sample was drawn), and may have affected the representativeness of the sample. Similarly, the refusal rate was 22%, which may have also affected sample representativeness, and the small sample size resulted in limited statistical power to detect associations. During screening for study enrollment, a higher than expected proportion (80.3%) reported no previous sex. This may reflect underreporting, and could relate to parental involvement in consent procedures. Biological markers would have allowed us to detect previous sex and mitigated some of the bias from relying on self-report. Unfortunately, currently available markers in vaginal fluids (Y-chromosome, semenogelin, prostate-specific antigen) do not reliably detect previous sex that occurred over 14 days prior to sample collection [47]. A serological marker of sexual exposure, such as herpes simplex virus type-2 (HSV-2) antibody [48] was not measured because of budget constraints, concerns that drawing a blood sample may have increased refusal rate, and because HSV-2 is not a gold standard for the detection of sexual debut [49]. Finally, self-administered vaginal swabs were collected rather than physician-collected endocervical specimens. The HPV genotypes detected therefore may not reflect cervical HPV genotypes [50]. However, it has been argued that vulvo-vaginal HPV infections may ascend to the cervix [7].

The proposed HPV vaccination program in Tanzania will target girls in primary school class 4, where the median age is 10 years, and a catch-up campaign in older girls has not thus far been proposed. This study provides useful evidence for policymakers in relation to the likely effectiveness of such a campaign. Overall, 80.3% of 15–16 year old girls reported no previous sex, 99.2% of whom did not have infection with either of the 2 high-risk HPV genotypes included in current HPV vaccines (HPV-16/18). The WHO recommend including older girls in catch-up campaigns if a significant proportion of girls are naive to HPV vaccine types [10]; our findings suggest that such a campaign in older girls may be efficacious in preventing infection with these HPV types.

The data we present strongly link prevalent HPV infection with reported intravaginal cleansing. Intravaginal cleansing may be a marker for undisclosed sexual activity in our population, but could alternatively be a novel mechanism for the nonsexual transmission of HPV. If this is the case, the identification of this potentially modifiable risk factor would be highly relevant for a country with one of the highest rates of cervical cancer in the world. Further research to confirm and understand the link between intravaginal cleansing and HPV is required and is currently underway in Mwanza.

## References

[1] Ferlay J, Shin H, Bray F, Forman D, Mathers C, Parkin DM (2010). GLOBOCAN 2008 v2.0, Cancer Incidence and Mortality Worldwide: IARC CancerBase No. 10 [Internet].

[2] Bouvard V, Baan R, Straif K (2009). A review of human carcinogens—Part B: biological agents. Lancet Oncol.

[3] de Sanjosé S, Diaz M, Castellsagué X (2007). Worldwide prevalence and genotype distribution of cervical human papillomavirus DNA in women with normal cytology: a meta-analysis. Lancet Infect Dis.

[4] Smith JS, Melendy A, Rana RK, Pimenta JM (2008). Age-specific prevalence of infection with human papillomavirus in females: a global review. J Adolesc Health.

[5] Giuliano A, Harris R (2002). Incidence, prevalence, and clearance of type-specific human papillomavirus infections: the young women's health study. J Infect Dis.

[6] Weaver B, Tu W, Shew M (2011). Acquisition of first human papillomavirus infection related to first vaginal intercourse and other sexually transmitted infections in adolescent women. J Adolesc Health.

[7] Winer R, Lee S, Hughes J (2003). Genital human papillomavirus infection: incidence and risk factors in a cohort of female university students. Am J Epidemiol.

[8] Hildesheim A, Herrero R (2007). Human papillomavirus vaccine should be given before sexual debut for maximum benefit. J Infect Dis.

[9] Rylander E, Ruusuvaara L, Almströmer MW, Evander M, Wadell G (1994). The absence of vaginal human papillomavirus 16 DNA in women who have not experienced sexual intercourse. Obstet Gynecol.

[10] WHO (2009). WHO position on HPV vaccines. Vaccine.

[11] Watson-Jones D, Baisley K, Ponsiano R (2012). Human papillomavirus vaccination in Tanzanian schoolgirls: cluster-randomized trial comparing 2 vaccine-delivery strategies. J Infect Dis.

[12] Andersson-Ellström A, Hagmar BM, Johansson B, Kalantari M, Wärleby B, Forssman L (1996). Human papillomavirus deoxyribonucleic acid in cervix only detected in girls after coitus. Int J STD AIDS.

[13] Kjaer SK, Chackerian B, van der Brule AJ (2001). High-risk human papillomavirus is sexually transmitted: evidence from a follow-up study of virgins starting sexual activity (intercourse). Cancer Epidemiol Biomarkers Prev.

[14] Fairley CK, Chen S, Tabrizi SN, Leeton K, Quinn MA, Garland SM (1992). The absence of genital human papillomavirus DNA in virginal women. Int J STD AIDS.

[15] Winer RL, Feng Q, Hughes JP, O'Reilly S, Kiviat NB, Koutsky LA (2008). Risk of female human papillomavirus acquisition associated with first male sex partner. J Infect Dis.

[16] Plummer ML, Ross DA, Wight D (2004). “A bit more truthful”: the validity of adolescent sexual behaviour data collected in rural northern Tanzania using five methods. Sex Transm Infect.

[17] Upchurch DM, Lillard LA, Aneshensel CS, Fang Li N (2002). Inconsistencies in reporting the occurrence and timing of first intercourse among adolescents. J Sex Res.

[18] Lees S, Cook C, Vallely A (2010). Comparison of sexual behavior data collected using a coital diary and a clinic-based interview during a microbicide pilot study in Mwanza, Tanzania. Sex Transm Dis.

[19] Doyle AM, Ross DA, Maganja K (2010). Long-term biological and behavioural impact of an adolescent sexual health intervention in Tanzania: follow-up survey of the community-based MEMA kwa Vijana Trial. PLOS Med.

[20] Plummer M, Wight D (2011). Young people's lives and sexual relationships in rural Africa: findings from a large qualitative study in Tanzania.

[21] Hildesheim A, Herrero R, Wacholder S (2007). Effect of human papillomavirus 16/18 L1 viruslike particle vaccine among young women with preexisting infection: a randomized trial. JAMA.

[22] Bosch FX, Qiao Y-L, Castellsagué X (2006). CHAPTER 2 The epidemiology of human papillomavirus infection and its association with cervical cancer. Int J Gynecol Obstet.

[23] Rombaldi RL, Serafini EP, Mandelli J, Zimmermann E, Losquiavo KP (2009). Perinatal transmission of human papilomavirus DNA. Virol J.

[24] Castellsagué X, Drudis T, Cañadas MP (2009). Human papillomavirus (HPV) infection in pregnant women and mother-to-child transmission of genital HPV genotypes: a prospective study in Spain. BMC Infect Dis.

[25] Marrazzo JM, Koutsky LA, Stine KL (1998). Genital human papillomavirus infection in women who have sex with women. J Infect Dis.

[26] Francis SC, Baisley K, Lees SS (2013). Vaginal practices among women at high risk of HIV infection in Uganda and Tanzania: recorded behaviour from a daily pictorial diary. PLOS ONE.

[27] Martin Hilber A, Hull TH, Preston-Whyte E (2010). A cross cultural study of vaginal practices and sexuality: implications for sexual health. Soc Sci Med.

[28] Lees S, Zalwango F, Andrew B (2014). Understanding motives for intravaginal practices amongst Tanzanian and Ugandan women at high risk of HIV infection: the embodiment of social and cultural norms and well-being. Soc Sci Med.

[29] McClelland RS, Lavreys L, Hassan WM, Mandaliya K, Ndinya-Achola JO, Baeten JM (2006). Vaginal washing and increased risk of HIV-1 acquisition among African women: a 10-year prospective study. AIDS.

[30] Tsai C, Shepherd B, Vermund S (2009). Does douching increase risk for sexually transmitted infections? A prospective study in high-risk adolescents. Am J Obstet Gynecol.

[31] Tarkowski TA, Koumans EH, Sawyer M (2004). Epidemiology of human papillomavirus infection and abnormal cytologic test results in an urban adolescent population. J Infect Dis.

[32] Winer RL, Hughes JP, Feng Q (2010). Detection of genital HPV types in fingertip samples from newly sexually active female university students. Cancer Epidemiol Biomarkers Prev.

[33] Strauss S, Sastry P, Sonnex C, Edwards S, Gray J (2002). Contamination of environmental surfaces by genital human papillomaviruses. Sex Transm Infect.

[34] Roden RBS, Lowy DR, Schiller JT (1997). Papillomavirus is resistant to desiccation. J Infect Dis.

[35] Watson-Jones D, Baisley K, Brown J (2013). High prevalence and incidence of human papillomavirus in a cohort of healthy young African female subjects. Sex Transm Infect.

[36] Dartell M, Rasch V, Kahesa C (2012). Human papillomavirus prevalence and type distribution in 3603 HIV-positive and HIV-negative women in the general population of Tanzania: the PROTECT study. Sex Transm Dis.

[37] Crucitti T, Jespers V, Mulenga C, Khondowe S, Vandepitte J, Buvé A (2011). Non-sexual transmission of Trichomonas vaginalis in adolescent girls attending school in Ndola, Zambia. PLOS ONE.

[38] Low N, Chersich MF, Schmidlin K (2011). Intravaginal practices, bacterial vaginosis, and HIV infection in women: individual participant data meta-analysis. PLOS Med.

[39] Brown D, Shew M, Qadadri B (2005). A longitudinal study of genital human papillomavirus infection in a cohort of closely followed adolescent women. J Infect Dis.

[40] Richardson H, Abrahamowicz M, Tellier P-P (2005). Modifiable risk factors associated with clearance of type-specific cervical human papillomavirus infections in a cohort of university students. Cancer Epidemiol Biomarkers Prev.

[41] Anemona A, Plummer M, Chilongani J (2002). The acceptability and quality of self-administered vaginal swabs among adolescents in rural Tanzania.

[42] Safaeian M, Kiddugavu M, Gravitt PE (2007). Comparability of self-collected vaginal swabs and physician-collected cervical swabs for detection of human papillomavirus infections in Rakai, Uganda. Sex Transm Dis.

[43] Ogilvie GS, Patrick DM, Schulzer M (2005). Diagnostic accuracy of self collected vaginal specimens for human papillomavirus compared to clinician collected human papillomavirus specimens: a meta-analysis. Sex Transm Infect.

[44] Gravitt PE, Schiffman M, Solomon D, Wheeler CM, Castle PE (2008). A comparison of linear array and hybrid capture 2 for detection of carcinogenic human papillomavirus and cervical precancer in ASCUS-LSIL triage study. Cancer Epidemiol Biomarkers Prev.

[45] Schiffman M, Wheeler CM, Dasgupta A, Solomon D, Castle PE (2005). A comparison of a prototype PCR assay and hybrid capture 2 for detection of carcinogenic human papillomavirus DNA in women with equivocal or mildly abnormal Papanicolaou smears. Am J Clin Pathol.

[46] UNICEF Country Information Tanzania http://www.unicef.org/infobycountry/tanzania_statistics.html.

[47] Minnis AM, Steiner MJ, Gallo MF (2009). Biomarker validation of reports of recent sexual activity: results of a randomized controlled study in Zimbabwe. Am J Epidemiol.

[48] Obasi A, Mosha F, Quigley M (1999). Antibody to herpes simplex virus type 2 as a marker of sexual risk behavior in rural Tanzania. J Infect Dis.

[49] Bastien S, Mason-Jones AJ, De Koker P, Mmbaga EJ, Ross DA, Mathews C (2012). Herpes simplex virus type 2 infection as a biomarker for sexual debut among young people in sub–Saharan Africa: a literature review. Int J STD AIDS.

[50] Belinson JL, Hu S, Niyazi M (2010). Prevalence of type-specific human papillomavirus in endocervical, upper and lower vaginal, perineal and vaginal self-collected specimens: implications for vaginal self-collection. Int J Cancer.

